# Leveraging Achromatic Component for Trichromat-Friendly Daltonization

**DOI:** 10.3390/jimaging11070225

**Published:** 2025-07-07

**Authors:** Dmitry Sidorchuk, Almir Nurmukhametov, Paul Maximov, Valentina Bozhkova, Anastasia Sarycheva, Maria Pavlova, Anna Kazakova, Maria Gracheva, Dmitry Nikolaev

**Affiliations:** 1 Institute for Information Transmission Problems of the Russian Academy of Sciences, 127051 Moscow, Russiapmaximov@iitp.ru (P.M.);; 2 Federal Research Center “Computer Science and Control” of the Russian Academy of Sciences, 119333 Moscow, Russia; d.p.nikolaev@smartengines.com; 3 Smart Engines Service LLC, 117312 Moscow, Russia

**Keywords:** achromatic component, color vision deficiency, contrast, daltonization, naturalness, subjective evaluation

## Abstract

Color vision deficiency (CVD) affects around 300 million people globally due to issues with cone cells, highlighting the need for effective daltonization methods. These methods modify color palettes to enhance detail visibility for individuals with CVD. However, they can also distort the natural appearance of images. This study presents a novel daltonization method that focuses on preserving image naturalness for both normal trichromats and individuals with CVD. Our approach modifies only the achromatic component while enhancing detail visibility for individuals with CVD. To compare our approach with the previously known anisotropic daltonization method, we utilize objective and subjective evaluations that separately assess visibility enhancement and naturalness preservation. Our findings indicate that the proposed method outperforms the anisotropic method in naturalness by over 10 times according to objective criteria. Subjective evaluations revealed that more than 90% of CVD individuals and 95% of trichromats preferred our method for its natural appearance. Although objective contrast metrics suggest inferior visibility enhancement, subjective evaluation indicates comparable performance: contrast improvement was observed in 65% of protan cases for our method versus 70% for the anisotropic method, with contrast deterioration in 18% versus 7%, respectively. Overall, our method offers superior naturalness while maintaining comparable detail discrimination.

## 1. Introduction

### 1.1. Background

Human color vision relies on three cone receptors in the retina, each sensitive to different wavelengths: long (L), medium (M), and short (S) [1]. Major color vision deficiencies (CVDs) include dichromacy, where one type of receptor is absent, and anomalous trichromacy, where receptor sensitivity deviates from the norm. Dichromacy is further classified into protanopia, deuteranopia, and the rare tritanopia, indicating the absence of L-, M-, and S-cones, respectively. CVDs affect about 300 million people globally [2].

Individuals with CVD struggle to distinguish specific color pairs [3], impacting their ability to perceive objects in their environment. This difficulty is pronounced when using visual display devices like smartphones, tablets, and televisions [4]. To assist these individuals, researchers have developed daltonization methods that improve the visibility of elements in color images for dichromats, making them distinguishable as they are for those with normal color vision (NCV) [5]. Techniques include artificial color palettes and contouring [6]. While some devices adjust interface colors for CVD, these adjustments are less effective for photos, and no single method has gained widespread acceptance [7].

Effective daltonization must also preserve image naturalness [5], defined as the match between the reproduced image and internal references, such as memory colors [8]. The stipulation of maintaining naturalness adds complexity to the daltonization. The method focused on preserving the image’s original colors was first introduced by Kuhn et al. [9]. Similar approaches have been devised by Hassan [10] and a research team led by Mao [11,12,13]. Regardless of the effort invested in preserving the natural appearance of recolored images for individuals with CVD, the color disparities between the recolored and original images remain noticeable to individuals with NCV [11]. Conversely, Choi et al. proposed a method that minimizes color differences between the recolored and original images, preserving naturalness for normal trichromats but not for CVD individuals [14]. Simultaneously maintaining naturalness for both groups is crucial for effective visual communication, enhancing social interaction through shared viewing experiences [15,16]. Techniques such as stereoscopic displays allow dichromats to view daltonized images while trichromats see the original version, but this requires specialized equipment [17]. Several software techniques aim to daltonize photos while preserving naturalness for both CVD and NCV individuals [13,18,19,20,21].

### 1.2. Paper Overview

To effectively preserve naturalness for both individuals with CVD and NCV, we utilize the human ability to decompose color perception of observed radiance into chromatic and achromatic components [22]. Currently, there are several definitions and terms referring to the achromatic component (lightness, value, brightness). The achromatic component, often referred to as lightness, is typically represented by the L* coordinate of the CIELAB system, although it is not entirely independent of chromatic components; see Section 5.2.

Adjusting the achromatic component of the image has a milder impact on object naturalness compared to altering chromaticity [21]. Several daltonization methods distinguish between chromatic and achromatic components to preserve naturalness [18,19,20,21]. Although these methods use different definitions of the achromatic component, they should be considered as belonging to the same achromatic approach. However, these methods often overlook the importance of preserving local contrast among neighboring pixels, which is crucial for human vision.

Our method aims to enhance local contrast and maintain naturalness for both dichromats and normal trichromats by modifying only the achromatic component. We conducted objective and subjective assessments to determine if substantial recovery of local contrast for dichromats can be achieved in this way. Moreover, we compared our method with Farup’s anisotropic daltonization approach, which alters more than just the achromatic component [23].

In our evaluations, we assessed contrast and naturalness separately. For the objective assessment of naturalness, we needed to modify the existing chromatic differences metric in such a way that it evaluated the chromatic component completely independently of the achromatic one. To achieve this, instead of using CIELAB color coordinates, we proposed using proLab coordinates [24].

The results show that our method significantly improves image contrast for CVD individuals while preserving naturalness for both CVD and normal trichromats. It shows effectiveness across different types and severity levels of CVD (protans and deutans, dichromats, and anomalous trichromats). Our algorithm is customized based on the specific type of CVD. The type of CVD is usually diagnosed using the Color Assessment and Diagnosis (CAD) method [25], but we validated the suitability of an express-test introduced by Maximov et al. for this purpose [26,27]. This simple test, which does not require expensive medical equipment or specialized print materials like Ishihara or Rabkin plates, can be self-administered by users.

In Section 2, we review different daltonization methods, noting their advantages and weaknesses. In Section 3, we describe the CVD simulation method we used. Section 4 describes our daltonization approach in detail. In Section 5, we introduce the metrics and dataset we used for the objective evaluation of our method. Section 6 describes the procedures for the subjective evaluation of our approach with both color-normal and color-deficient observers. We compare our method with anisotropic daltonization in Section 7. In Section 8, we conclude our work and specify a future direction.

### 1.3. Contribution

The contributions of this paper are as follows:

1. A novel daltonization method that preserves local contrast and naturalness for both dichromats and trichromats. Unlike methods that fail to maintain trichromatic naturalness, our approach enables shared content viewing by both dichromats and trichromats.
2. A public dataset designed to facilitate future comparisons of daltonization methods within a unified benchmark. The dataset includes a collection of images and the results of their daltonization using two evaluated methods for two types of dichromacy, as well as the outcomes of subjective quality assessments of daltonization for each image.
3. Improvement of a previously known objective metric for assessing naturalness based on chromatic differences in the proLab color space. The revised metric is sensitive to any changes in color hue, which was not achieved in earlier approaches.
4. Validation of the CVD express-test for diagnosing types of dichromacy, previously proposed by one of the co-authors. Unlike the widely used CAD method, the investigated CVD express-test does not require specialized equipment or laboratory personnel. This is particularly important for daltonization methods integrated into personal devices (such as smartphones), as they allow users to independently assess the characteristics of their color vision and activate a compensatory mode if necessary.

## 2. Related Works

In this section, we review methods aimed at preserving naturalness for both dichromats and normal trichromats, and their modifications.

The method presented in [13] can be regarded as a method that aims to maintain naturalness for both dichromats and normal trichromats. Within the framework of this method, first, an image simulating the loss of contrast experienced by dichromats is generated. This image is then daltonized, aiming to preserve naturalness for dichromats. Only at the final step, to achieve naturalness for trichromats, is a component of the original image—indistinguishable to dichromats—added to the processed image. The authors indicate that this former operation fails to achieve its intended goal.

In the study by Chen et al. [28], the authors proposed a neural network-based daltonization method termed CVD-Swin. This approach utilizes a loss function defined as a weighted sum of two components: one aimed at preserving image naturalness and the other at restoring contrast typically lost in dichromatic vision. However, the study did not establish the optimal weighting between these two objectives.

In the following, methods that manipulate only the achromatic component of the image are considered.

**Figure 1:**
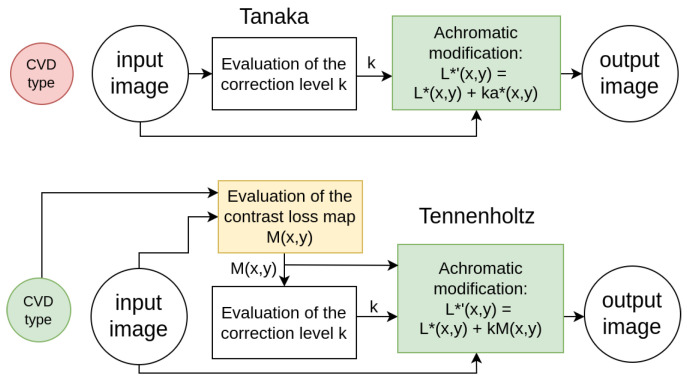
Diagrams of Tanaka and Tennenholz methods.

Tanaka’s method [21] augments each pixel’s achromatic component ($L^{*}\left(x,y\right)$ component of the CIELAB image) with its chromatic component ($a^{*}\left(x,y\right)$ of the CIELAB image) using an optimized unified coefficient for individual input images (Figure 1). The main disadvantage of Tanaka’s method is that it does not take into account the type of CVD, which leads to highly inaccurate restoration of lost details.

Tennenholtz’s method [20] computes a mean contrast loss map using each pixel’s neighborhood and modifies the achromatic component based on this map (Figure 1). In this method, as highlighted by Meng and Tanaka [21], the visibility of elements within the image is enhanced by emphasizing the contours of the objects. Restoring object contours instead of the objects themselves allows partial recovery of the informational content of the original image, however, it alters its appearance.

To restore not the contours of objects but the objects themselves, several methods utilize the concept proposed by Socolinsky and Wolff [29]. In this concept, local contrast is determined by the Di Zenzo structural tensor, from which a target vector field of original contrast is derived. The resulting image is adjusted as a whole to make its gradient field as close as possible to the target. We consider the approach proposed by Socolinsky and Wolff to be the most promising, and the methods based on it will be examined further.

**Figure 2:**
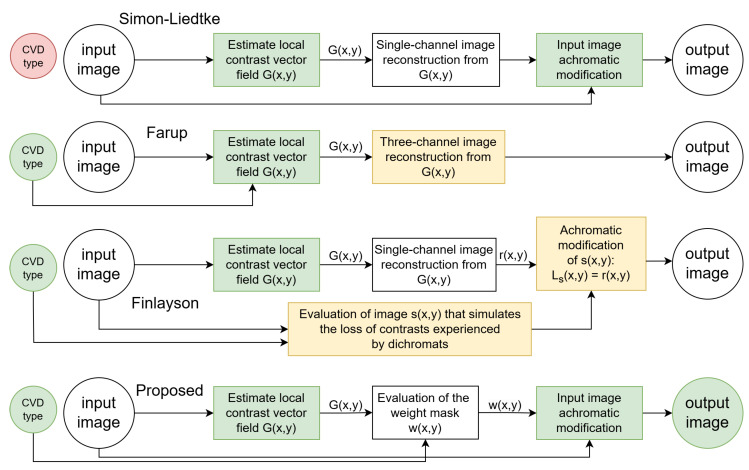
Diagrams of Simon-Liedtke method, Farup method, Finlayson method, and proposed method.

Simon-Liedtke and Farup [19] introduced such a method that ensures naturalness by processing only the achromatic component. However, a drawback of the method proposed in [19] is that the contrast preservation does not account for CVD type, utilizing a contrast-preserving RGB-to-grayscale transformation (Figure 2).

The subsequent research by the Farup group took a different direction [23,30]. In his 2020 work [23], Farup presented an anisotropic daltonization method aimed at maintaining local contrast specifically lost in observers with CVD (Figure 2). However, this method does not ensure naturalness preservation as it involves modifications beyond the achromatic component.

Finlayson’s group also discussed a daltonization method based on Socolinsky and Wolff’s approach, employing the POP image fusion technique to simplify the original method [31]. The paper suggests compressing the original RGB channels into one using POP and substituting the achromatic component of the image. In Finlayson’s method, an image is constructed that simulates the loss of contrast experienced by dichromats, with modifications applied to the achromatic component of this image (Figure 2). The resulting image is unnatural for trichromats.

To address the identified shortcomings of the related methods, we propose a novel daltonization technique. Our method maintains naturalness by modifying only the achromatic component of the image and establishes an optimization problem similar to Socolinsky and Wolff’s approach [29] to retain local contrast (Figure 2). Preliminary results, without a detailed description and comprehensive testing, were reported in [32].

## 3. Simulation Method

To develop a daltonization algorithm, it is crucial to model color images as perceived by individuals with CVD. This is achieved through simulating color perception for dichromats. In this study, we employed the dichromat simulation algorithm proposed by Viénot et al. [33]. We did not use any other algorithm to model anomalous trichromacy.

The following basic requirement is imposed on dichromat color perception simulators: each set of colors indistinguishable to a dichromat must be displayed by the simulator in one color from this set, but which one is not regulated. Clearly, there can be an infinite variety of approaches to simulating dichromatic vision. They vary in the representation of the “blind” channel in the simulated image—the channel that corresponds to the type of missing cones (L for protanopia, M for deuteranopia). Typically, the specific color assigned to represent a set of indistinguishable colors is chosen to satisfy some additional requirement, such as preserving colors that are assumed to appear the same to dichromatic and normal observers. The most straightforward mathematically are simulators employing linear transformations of color vectors: the signal of the “blind” channel becomes a linear combination of the signals from the other two channels. Note that the well-known Brettel’s simulator is only piecewise linear since it uses two half-planes as a model of a dichromat color space [34].

**Figure 3:**
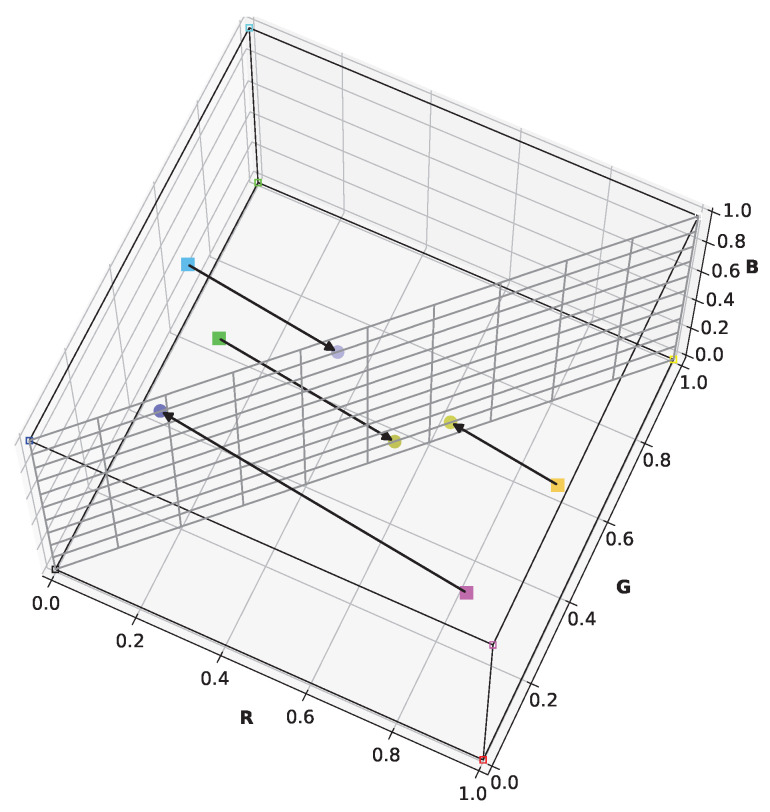
Simulation of protanopic perception for four colors in linear RGB color space by Viénot’s linear simulation. Solid colored squares indicate original colors, and colored circles indicate simulated colors. Black arrows show the transformation of colors in the course of the simulation.

Viénot’s simulation retains blue, yellow, and grayscale in the simulation image—colors distinguishable by protanopes and deuteranopes. Furthermore, it utilizes almost the entire display’s color range. Figure 3 illustrates the simulation of certain arbitrary colors for a protanope. It can be seen that the plane representing the dichromat color space contains the black, white, blue, and yellow points of the RGB cube. Unlike Brettel’s algorithm, this linear approach streamlines its integration into the daltonization algorithm. These aspects are crucial for visually evaluating contrast loss in dichromats and achieving contrast restoration through daltonization.

Since Viénot et al. [33] developed their simulations for a display with the same primaries and reference white as the sRGB standard uses, we were able to use the matrix for converting linear RGB values to trichromat cone responses (LMS) from that paper without change. When applying the algorithm to displays with different color gamuts, recalculating the matrix is straightforward using the authors’ proposed methodology. We also utilized the authors’ simulation matrices for protanope and deuteranope perception without making any alterations.

Because both linear RGB-to-LMS conversion and dichromat simulation in the LMS color space are linear operations, we can replace the individual sequential matrix–vector multiplications with a single multiplication of the product of the corresponding matrices by the color vector. Following the notation of Viénot et al. [33] we can write the following for a protanope:

(1) $$\left(\begin{array}{c} R_{p}\\ G_{p}\\ B_{p} \end{array}\right)={\left(RGB\_\mathrm{to}\_LMS\right)}^{-1}\left(LMS\_\mathrm{to}\_L_{p}M_{p}S_{p}\right)\times \left(RGB\_\mathrm{to}\_LMS\right)\left(\begin{array}{c} R\\ G\\ B \end{array}\right),$$

where ${\left(R_{p},G_{p},B_{p}\right)}^{T}$ is the protanope simulation of the color vector ${\left(R,G,B\right)}^{T}$, (RGB_to_LMS) is the matrix converting linear RGB values to LMS values, and $\left(LMS\_\mathrm{to}\_L_{p}M_{p}S_{p}\right)$ is the protanope simulation matrix in the LMS color space. A similar equation can be written for the deuteranope simulation.

Thus, the simulation is a linear transformation described by the following matrix equation:

(2) $$\vec{c_{s}}=\hat{D}\;\vec{c},$$

where $\vec{c}$ represents the original color vector, $\vec{c_{s}}$ is the color vector in the simulated image, and $\hat{D}$ is the simulation matrix. The following matrices were used for simulating the perception of protanopes and deuteranopes:

(3) $${\hat{D}}^{P}={\left[\begin{array}{ccc} 0.1124 & 0.8876 & 0\\ 0.1124 & 0.8876 & 0\\ 0.0040 & -0.0040 & 1 \end{array}\right]}_{linRGB},\;\;{\hat{D}}^{D}={\left[\begin{array}{ccc} 0.2928 & 0.7072 & 0\\ 0.2928 & 0.7072 & 0\\ -0.0223 & 0.0223 & 1 \end{array}\right]}_{linRGB}.$$

**Figure 4:**
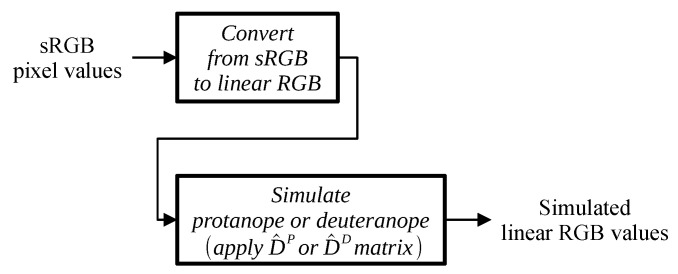
Simulation scheme.

Conversion of the image pixel values to the simulated linear RGB values for a protanope or deuteranope is illustrated in Figure 4.

## 4. Achromatic Contrast-Preserving Daltonization

As mentioned in Section 2, daltonization methods aim to preserve local contrast. The formalization of the local contrast of a single-channel image is a gradient. For multi-channel images, a pseudo-gradient was introduced by Socolinsky and Wolff [29]. Following them, we define a multi-channel image as a rectangle $\Omega \in R^{2}$ together with a map $u:\Omega \to P^{n}$, where $P^{n}$ denotes *n*-dimensional photometric space. The pseudo-gradient is based on the structural tensor *S* defined by Di Zenzo [35],

(4) $$S\left(i,j\right)=\nabla \vec{u}{\left(i,j\right)}^{T}\nabla \vec{u}\left(i,j\right),$$

where $\nabla \vec{u}\left(i,j\right)$ denotes the Jacobian matrix of image $\vec{u}$ at point (i,j):

(5) $$\nabla \vec{u}\left(i,j\right)=\left[\begin{array}{cc} \frac{\partial {\vec{u}}^{1}\left(i,j\right)}{\partial i} & \frac{\partial {\vec{u}}^{1}\left(i,j\right)}{\partial j}\\ \vdots & \vdots \\ \frac{\partial {\vec{u}}^{n}\left(i,j\right)}{\partial i} & \frac{\partial {\vec{u}}^{n}\left(i,j\right)}{\partial j} \end{array}\right].$$

The pseudo-gradient is represented by the primary eigenvalue $\lambda_{\max}$ along with the associated eigenvector ${\vec{e}}_{\max}$ of the structural tensor *S*:

(6) $${\tilde{\nabla }}_{\pm }\;\vec{u}\left(i,j\right)=\pm \sqrt{\lambda_{\max}}\;{\vec{e}}_{\max}.$$

For selecting the sign of the pseudo-gradient in Equation (6), several solutions exist [36,37,38]. One straightforward but effective method involves choosing directions along the gradients of the integral image formed by summing the channels of the original image at each pixel [29]. The pseudo-gradient with the selected sign is denoted as $\tilde{\nabla }$.

Initially, a local contrast-preserving paradigm was proposed for the multi-channel image visualization problem, where it is necessary to reduce the number of input channels from two or more to one while preserving information important for the human visual system [29,39]. Next, the contrast-preserving visualization approach is briefly discussed. Then we use this approach for daltonization. The key aspect of the Socolinsky and Wolff contrast-preserving paradigm is the image reconstruction using known contrast. Namely, estimation of the resulting grayscale image $u^{*}$ during optimization so that its gradient $\nabla u^{*}$ matches the pseudo-gradient $\tilde{\nabla }{\vec{u}}_{0}$ as much as possible:

(7) $$u^{*}=\arg\min_{u}\underset{\Omega }{\;\int \int \;}{∥\nabla u-\tilde{\nabla }{\vec{u}}_{0}∥}_{2}^{2}d\Omega ,$$

where ${\vec{u}}_{0}$ is the input image and ${∥\cdot ∥}_{2}$ indicates the Euclidean norm. The optimization criterion in Formula (7) is commonly referred to as linear [37,40].

According to the Weber–Fechner law, the psychophysical significance lies in the relative values of stimulus intensities. In the current context, this law becomes apparent as gradient errors are clearly distinguishable in monotonic areas of the image, while in non-monotonic areas, the same absolute errors remain unnoticed. The linear criterion (7) does not consider this perceptual effect, potentially resulting in the emergence of unwanted gradients (halo) in the final image [41]. To address this, Sokolov et al. proposed to replace the linear criterion with a non-linear alternative [37,40]:

(8) $$u^{*}=\arg\min_{u}\underset{\Omega }{\;\int \int \;}\frac{∥\nabla u-\tilde{\nabla }{\vec{u}}_{0}{∥}_{2}^{2}}{{∥\nabla u∥}_{2}^{2}+{∥\tilde{\nabla }{\vec{u}}_{0}∥}_{2}^{2}+\epsilon^{2}}d\Omega ,$$

where ϵ is the regularization constraint. It has been shown that contrast-preserving visualization with the Sokolov criterion outperforms alternative approaches [42]. To date, we are not aware of any other non-linear criteria. Now let us return to the daltonization, taking functional (8) as a starting point.

Similar to the visualization methods, daltonization methods should be based on channel number reduction: to improve visibility of image elements for individuals with CVD, one needs to model the contrast loss they experience. This is achieved using the simulation method, which for a given CVD type reduces one channel that corresponds to the type of missing cones. Unlike the visualization approach, daltonization methods return an image with the same number of channels as the input image. In our daltonization algorithm, we use the described visualization approach with several modifications.

First, we replace the resulting single-channel image *u* with a three-channel daltonized image $\vec{u}$. The gradient of a single-channel image ∇u is replaced with the pseudo-gradient of a three-channel image $\tilde{\nabla }\vec{u}$. Second, we note that in the denominator of the non-linear Sokolov criterion, the contribution of the contrast of the resulting image ${∥\nabla u∥}_{2}^{2}$ and the contrast of the original image $∥\tilde{\nabla }{\vec{u}}_{0}{∥}_{2}^{2}$ are equal. However, in the visualization, as well as in the daltonization, this is not the case. The input image contains reference contrasts to which the output image should be as close as possible, and not vice versa. If ${∥\nabla u∥}_{2}^{2}\to \infty$ while $∥\tilde{\nabla }{\vec{u}}_{0}{∥}_{2}^{2}\to 0$, the criterion value should tend to infinity; which is not satisfied for criterion (8).

Combining the above, we propose the following non-linear criterion for daltonization:

(9) $${\vec{u}}^{*}=\arg\min_{\vec{u}}\underset{\Omega }{\;\int \int \;}\frac{∥\tilde{\nabla }\left[\hat{D}\vec{u}\right]-\tilde{\nabla }{\vec{u}}_{0}{∥}_{2}^{2}}{∥\tilde{\nabla }{\vec{u}}_{0}{∥}_{2}^{2}+\epsilon^{2}}d\Omega ,$$

where $\hat{D}$ is the simulation matrix. This criterion, on the one hand, retains the advantage of the Sokolov criterion, that is, it prevents the appearance of a halo; on the other hand, it is asymmetrical in favor of the reference image.

The problem statement (9) is focused on preserving local contrast, but so far does not take into account the preservation of naturalness for both dichromats and trichromats. To highlight boundaries invisible to dichromats, we modulate the achromatic component of the pixels by multiplying the values of $\vec{u_{0}}$ by a certain weight *w*, calculated for each pixel:

(10) $$\vec{u}=w^{*}\;{\vec{u}}_{0},\;w^{*}=\arg\min_{w}\underset{\Omega }{\;\int \int \;}\frac{∥\tilde{\nabla }\left[\hat{D}\;w\;{\vec{u}}_{0}\right]-\tilde{\nabla }{\vec{u}}_{0}{∥}_{2}^{2}}{∥\tilde{\nabla }{\vec{u}}_{0}{∥}_{2}^{2}+\epsilon^{2}}d\Omega ,$$

where ${\vec{u}}_{0}$ denotes the original image, *w* is the weight mask, and $\vec{u}$ is the resulting daltonized image.

We replace the pseudo-gradient with the Euclidean norm of the difference of two color vectors in pixels with coordinates *p* and *q*. In the current study we consider *p* and *q* as close neighbors: for each p=(x,y) the pair q=(x+1,y) and q=(x,y+1) is considered. Potentially, it might be useful to increase the size of the neighborhood as human vision perceives local contrast at different scales, depending on the angular resolution of the observed image [43]. Finally, the discretized problem (10) takes the following form:

(11) $$w^{*}=\arg\min_{w}\sum_{p,q}\frac{{\left(∥\hat{D}\;w_{p}{\vec{u}}_{p}-\hat{D}\;w_{q}{\vec{u}}_{q}{∥}_{2}-{∥{\vec{u}}_{p}-{\vec{u}}_{q}∥}_{2}\right)}^{2}}{∥{\vec{u}}_{p}-{\vec{u}}_{q}{∥}_{2}^{2}+\epsilon^{2}},$$

The Euclidean norm, unlike the pseudo-gradient, allows for an increased neighborhood without any modifications in the problem statement.

Functional (11) is non-convex and thus difficult to optimize. In this regard, we make several simplifications. First, we focus solely on the numerator of the original functional (11):

(12) $$\begin{array}{c} w^{*}=\arg\min_{w}\sum_{p,q}{\left(∥w_{p}\hat{D}\;{\vec{u}}_{p}-w_{q}\hat{D}\;{\vec{u}}_{q}{∥}_{2}-{∥{\vec{u}}_{p}-{\vec{u}}_{q}∥}_{2}\right)}^{2}. \end{array}$$

Second, we employ variable substitutions of pairs $w_{p}$, $w_{q}$ and ${\vec{u}}_{p}$, ${\vec{u}}_{q}$ by their difference and mean values:

(13) $$\left\{\begin{array}{cc} \Delta w_{p,q}=w_{p}-w_{q} & \\ {\bar{w}}_{p,q}=\frac{1}{2}\left(w_{p}+w_{q}\right) \end{array}\right.,\;\left\{\begin{array}{cc} \Delta {\vec{u}}_{p,q}={\vec{u}}_{p}-{\vec{u}}_{q} & \\ {\bar{\vec{u}}}_{p,q}=\frac{1}{2}\left({\vec{u}}_{p}+{\vec{u}}_{q}\right) \end{array}\right.,$$

and assume that the mean mask value between adjacent pixels remains constant: ${\bar{w}}_{p,q}=const$. By substituting the original variables with new ones and plugging them into (12), we obtain a quadratic equation for a $\Delta w_{p,q}$:

(14) $$∥\Delta w_{p,q}\hat{D}\;{\bar{\vec{u}}}_{p,q}+\bar{w}\hat{D}\;\Delta {\vec{u}}_{p,q}{∥}_{2}^{2}-{∥\Delta {\vec{u}}_{p,q}∥}_{2}^{2}=0.$$

Equation (14) is solved independently for each pair of pixels *p* and *q*, where each pixel of the image is alternately selected as p=(x,y) and generates two pairs: with q=(x+1,y) and with q=(x,y+1).

To choose between the two solutions of (14), the guiding differences are used. The guiding differences are calculated using the integral image formed by summing the channels of the original image, as in [29]. If for a given pair p,q the guiding difference is greater than zero, then the largest of the two solutions is chosen; if less, then the smallest.

Due to the variable substitutions, the solution of the simplified problem (14) is not the pixel weight $w_{p},w_{q}$ values but their differences $\Delta w_{p,q}$. This brings us back to the problem of image reconstruction using known contrast. To this purpose, we use the following non-linear criterion with the above-proposed denominator modifications (as in criterion (9)):

(15) $$w^{*}=\arg\min_{w}\sum_{p,q}\frac{{\left(\left(w_{p}-w_{q}\right)-\Delta w_{p,q}\right)}^{2}}{\Delta w_{p,q}^{2}+\epsilon^{2}}.$$

This problem is solved using the Adam optimizer from PyTorch v2.6.0.

Multiplying by the weights obtained from (15) may cause the daltonized image $\vec{u}$ values to exceed the RGB displayable range, which cannot be reproduced correctly on standard displays. This problem is known as tone mapping and is being researched in the field of high-dynamic-range (HDR) imaging, where it is necessary to compress the HDR signal for visualization on varied low-dynamic-range output devices such as regular displays [44,45]. In this study, we employ a tone-mapping technique utilizing autocontrast with a threshold set at the 0.98 quantile [46].

**Figure 5:**
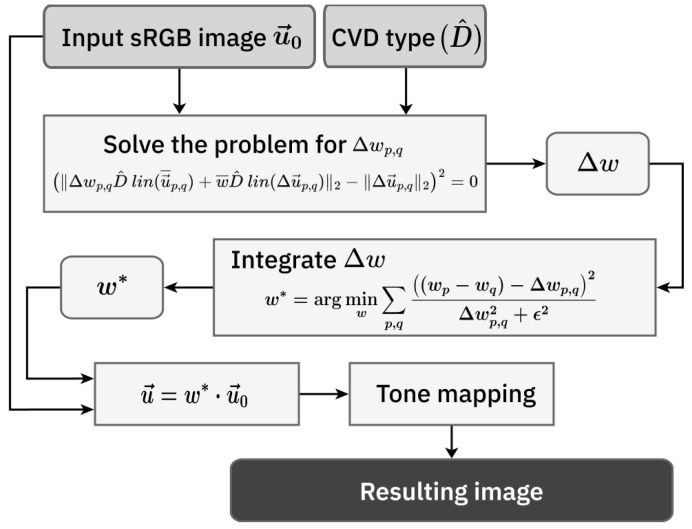
Scheme of the proposed method.

The overall framework of the proposed method is illustrated in Figure 5. The source code of the proposed method is available for download: https://github.com/iitpvisionlab/achromatic-daltonization (accessed on 24 June 2025).

## 5. Objective Evaluation

Our daltonization involves two subproblems: enhancing element visibility and maintaining naturalness. To objectively assess daltonization methods, we employ metrics that individually evaluate visibility enhancement and naturalness preservation, rather than complex image quality metrics that obscure the contribution of specific components.

### 5.1. Contrast Preservation

For evaluating contrast preservation, we utilize the RMS (root mean square) metric, which computes a root-mean-square difference between pairs of images across corresponding neighborhoods [47]. This metric was proposed to “capture local differences in color contrast between pairs of images” and is widely used in daltonization studies [11,47,48].

(16) $$\begin{array}{c} \mathrm{RMS}\left(u_{ref},u_{test}\right)=\frac{1}{\left|I\right|}\sum_{i\in I}\sqrt{\frac{1}{|\Theta_{i}|}\sum_{j\in \Theta_{i}}{\left(\frac{∥{\vec{u}}_{i}^{\;ref}-{\vec{u}}_{j}^{\;ref}{∥}_{2}-{∥{\vec{u}}_{i}^{\;test}-{\vec{u}}_{j}^{\;test}∥}_{2}}{160}\right)}^{2}}, \end{array}$$

where $u_{ref}$ and $u_{test}$ correspond to the original and resulting images in CIELAB coordinates, ${\vec{u}}_{i}^{\;ref}$ and ${\vec{u}}_{i}^{\;test}$ are the corresponding values in the *i*-th pixel, *I* is the set of pixels under consideration, $\Theta_{i}$ is the set of neighboring pixels for the *i*-th pixel within *I*, |·| denotes the cardinality of a set, and ${∥\cdot ∥}_{2}$ denotes the Euclidean norm. According to Machado and Oliveira [47], “The constant 160 in the denominator keeps the resulting value in the [0, 1] range.” In the experiments detailed in Section 7, set *I* was established using a regular grid with 10-pixel intervals. A total of 1000 pixel coordinates within set $\Theta_{i}$ were sampled using a two-dimensional normal distribution centered at pixel *i*, with a σ of 25% relative to the linear size of the evaluated image.

### 5.2. Naturalness Preservation

Psychophysics studies indicate that naturalness perception is affected by many factors, such as color, sharpness, contrast, blur, glare imitation, and shadow detail reproduction [49,50]. In the current work, we rely on the experimental evidence suggesting that observers tend to discount achromatic component changes while judging naturalness [8,51,52]. Therefore, we employ chromatic difference (CD) to assess naturalness preservation. CD, introduced in several studies including [10,11,13,48,53], modifies the color difference metric by disregarding variations in lightness [53].

Wang et al. [11] computed the CD to evaluate naturalness in their study using the following formula:

(17) $$\begin{array}{c} CD_{Lab}\left(u_{ref},u_{test}\right)=\frac{1}{N}\sum_{i=1}^{N}\sqrt{{\left(a_{i}^{test}-a_{i}^{ref}\right)}^{2}+{\left(b_{i}^{test}-b_{i}^{ref}\right)}^{2}}, \end{array}$$

where *N* is the total pixel count, $u_{ref}$ is the reference image, $u_{test}$ is the test image, and $a^{test}$, $a^{ref}$, $b^{test}$, $b^{ref}$ are the CIELAB chromaticity coordinates of the test and reference images, respectively. The CD metric operates under the assumption that these coordinates portray chromaticity. However, we demonstrate that this assumption is incorrect.

As per the ICC.2:2019 specification [54], the conversion from XYZ coordinates to CIELAB coordinates is performed using the following formula:

(18) $$\begin{array}{cc} \; & L=116f\left(\frac{Y}{Y_{n}}\right)-16,\\ \; & a=500\left(f\left(\frac{X}{X_{n}}\right)-f\left(\frac{Y}{Y_{n}}\right)\right),\\ \; & b=200\left(f\left(\frac{Y}{Y_{n}}\right)-f\left(\frac{Z}{Z_{n}}\right)\right), \end{array}$$

where

(19) $$\begin{array}{cc} f(t) & =\left\{\begin{array}{cc} \sqrt[{3}]{t} & \mathrm{when}\;t>{\left(\frac{6}{29}\right)}^{3}\\ \frac{841}{108}t+\frac{4}{29} & \mathrm{when}\;t\leq {\left(\frac{6}{29}\right)}^{3} \end{array}\right.. \end{array}$$

**Figure 6:**
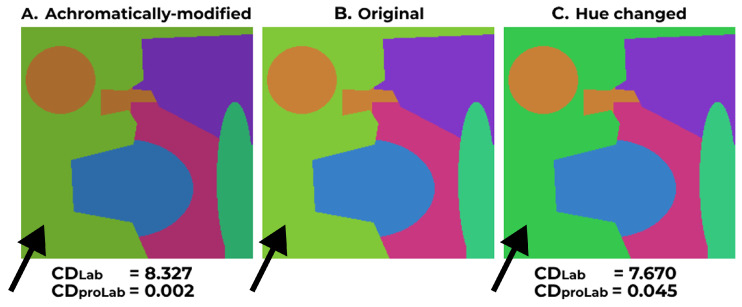
Illustration of difference between $CD_{Lab}$ and $CD_{proLab}$; (**A**) shows the image perceived as achromatically modified version of (**B**); (**B**) shows the original image, taken from [41]; (**C**) shows the image perceived as hue-altered version of (**B**). Note the left green regions in each image, indicated by black arrows.

Note that multiplying the *X*, *Y*, and *Z* coordinates by any scalar does not affect the chromaticity in terms of the CIE xy chromaticity diagram [55]. However, according to Equation (18), the values of *a* and *b* would alter. That is why CIELAB coordinates are poorly suited for chromaticity difference assessment. This nuance is demonstrated in Figure 6. CD values, calculated in CIELAB using Formula (17), imply that Figure 6C is closer in chromaticity to the original Figure 6B than Figure 6A. This difference in CD values contradicts the visual perception: Figure 6C exhibits noticeable hue distortion within the left green region.

To ensure accurate evaluation of chromaticity preservation, we suggest using chromaticity coordinates derived from proLab [24]. This color coordinate system is derived through a 3D projective transformation of CIE XYZ. For chromaticity difference estimation, it is crucial that after the projective transform, the color coordinates a^+^ and b^+^ are divided by L^+^:

(20) $$\tilde{a}=\frac{a^{+}}{L^{+}},\;\tilde{b}=\frac{b^{+}}{L^{+}}.$$

This renders the CD metric based on proLab coordinates $\tilde{a}$ and $\tilde{b}$ invariant to achromatic scaling, unlike the Lab coordinates *a* and *b*:

(21) $$\begin{array}{c} CD_{proLab}\left(u_{ref},u_{test}\right)=\frac{1}{N}\sum_{i=1}^{N}\sqrt{{\left({\tilde{a}}_{i}^{test}-{\tilde{a}}_{i}^{ref}\right)}^{2}+{\left({\tilde{b}}_{i}^{test}-{\tilde{b}}_{i}^{ref}\right)}^{2}}. \end{array}$$

According to $CD_{proLab}$ in Figure 6, the achromatically modified Figure 6A is closer in chromaticity to the original Figure 6B than the hue-altered Figure 6C, which aligns with visual perception.

### 5.3. Dataset

**Figure 7:**
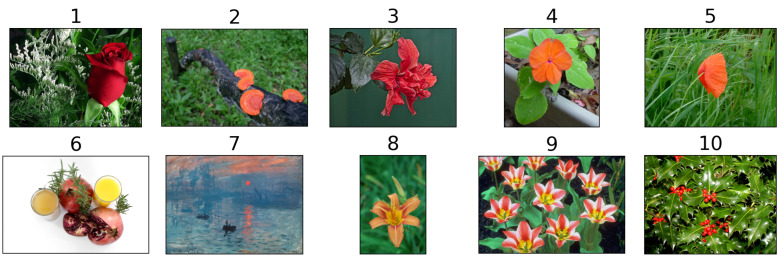
Collection of images utilized to assess daltonization methods.

To evaluate the proposed method, we curated a dataset of 10 colorful images sourced from relevant publications [23,30,56]. Datasets of a similar size are typical in daltonization studies: 16 images in [30], 10 images in [13], 14 images in [23], 8 images in [57], and 10 images in [12]. For images from [23,30], we retrieved the original high-resolution images through a reverse image search. Each image in the dataset features a distinct red–green color contrast between an object and its background, presenting challenges for individuals with deuteranopia and protanopia. Our image selection process was informed by corresponding simulations. Figure 7 illustrates the compiled image set used for testing the daltonization methods. The dataset is available for download via the following link: https://zenodo.org/records/14170170 (accessed on 24 June 2025).

## 6. Subjective Evaluation

Relying solely on quantitative metrics for assessments does not offer insight into how individuals with CVD perceive the results produced by the proposed tools [7]. Therefore, we conducted human studies involving individuals with CVD to validate our method. The same dataset was utilized for both subjective and objective evaluations.

### 6.1. Diagnosis of Color Vision Deficiency

Prior to the human studies, all participants underwent color vision assessment using the Color Assessment and Diagnosis (CAD) test [25]. The CAD system with an Eizo monitor CS2420 was calibrated and supplied by City Occupational LTD (London, UK); for calibration details, see [58]. CAD allows reliable and accurate identification of protans and deutans.

Additional separation into pure dichromacy and anomalous trichromacy (e.g., separation of protans into protanope and protanomalous subjects) requires additional analysis beyond the standard functionality of the system. To identify pure protanopes and deuteranopes, we inspected threshold plots retrieved from the CAD system. On the plot, we checked whether the observer’s threshold endpoints fully reached the edges of the monitor gamut. Subjects whose threshold endpoints lay on the monitor gamut boundaries were classified as putative dichromats (protanopes or deuteranopes). This method is based on the observation described in [25]. The authors of the review state: “subjects who hit the phosphor limits in the CAD test tend to also accept ‘any’ RG mixture as a match to the monochromatic yellow field in the anomaloscope test.”

### 6.2. Participants

A total of 17 individuals participated in our studies, comprising 3 normal trichromats, 6 individuals with protan deficiency (3 protanopes and 3 protanomalous trichromats), and 8 individuals with deutan deficiency (1 deuteranope and 7 deuteranomalous trichromats). The relatively small number of subjects is typical of daltonization studies and is due to the difficulty of finding and diagnosing people with CVD: 30 participants in [30], 12 participants in [13], 10 participants in [57], and 10 participants in [12]. In our study, the age of participants with CVD ranged from 21 to 63, averaging 35 years, with 50% of the group aged between 21 and 26.

Volunteers with CVD were recruited based on self-reported color perception issues in daily life to partake in diagnostics and experiments. All participants were male. As per the CAD test, their RG thresholds varied from 13 to 30 CAD units, categorizing them as having severe RG color deficiency or CV5 [25], comprising candidates with RG threshold values > 12 CAD units and YB threshold values within the age-related norm.

Additionally, two individuals exhibited both deutan/protan and mild tritan CVD (YB thresholds 2.25–2.57 CAD units), suggesting a possible acquired deficiency. However, only their deutan/protan CVD was considered for the results.

Except for one deuteranomalous trichromat, all participants underwent the CVD express-test to determine the type of CVD, and the corresponding results are presented in Supplementary Table S1.

### 6.3. Subjective Evaluation Methodology for Daltonization Method

We compared our proposed method with Farup’s anisotropic daltonization technique [23], which has publicly available source code. The participants were presented with two questions regarding contrast and naturalness for each of the 10 images in the test set. In the instructions, the contrast was referred to as “distinguishability of objects”. The definition of naturalness given in the introduction implies a subjective assessment from memory. However, the methodology based on such a definition suggests complex instructions and is not suitable for images of objects that may have different colors not only after daltonization but also in reality (e.g., flowers). Therefore, instead of providing complex instructions regarding memory, participants were suggested to evaluate the processed image in terms of its similarity to the explicit reference image.

When evaluating the naturalness of daltonization, only degradation is possible, and the task is to assess the degree of this degradation. Therefore, it is reasonable to use the triple-stimulus method with explicit reference, in which three images are simultaneously displayed on the screen: a reference image and two images being evaluated. This methodology involves direct comparison of two methods and provides easily interpretable results. When evaluating contrast, it is possible to observe not only degradation but also improvement relative to the reference. To not only compare algorithms but also accurately position them relative to the reference, the triple-stimulus methodology is unsuitable. For contrast evaluation, it is necessary to use the double-stimulus method with explicit reference, in which two images are displayed on the screen: the reference image and the image being evaluated. This methodology allows algorithms to be assessed independently, which also enables the comparison of results from different experiments conducted with various algorithms on the same dataset.

Combining all the considerations mentioned above, the following instructions were formulated:

1. “Which of the two images is more similar to the reference image?” Three images were displayed: a reference image and two test images (processed by both our method and the anisotropic daltonization technique). Answer options included choosing one of the images or selecting “Not sure”.
2. “Evaluate the distinguishability of objects compared to the reference. Disregard colors.” Two images were shown: a reference image and a single test image. Participants assessed the test image among four options: “Distinguishability on the test image is better than on the reference one”, “Distinguishability on the test image is worse than on the reference one”, “Approximately the same”, or “Not sure”.

No additional instructions were given. There were no restrictions on response time. Participants selected their answers using a computer mouse. Images were shown on a computer display with a resolution of 1920 by 1080 and a diagonal of 27 inches. The distance between the monitor and the observer was approximately 60 cm. Thus, the average angular size of the images was 8 degrees vertically, 12 degrees horizontally. During the assessment of both daltonization methods, participants with CVD viewed the test images, processed based on their CVD type. Participants with NCV (normal trichromats) were presented randomly with test images processed for protanopes or deuteranopes. Informed consent was obtained from all participants in the study.

### 6.4. CVD Express-Test

The contrast loss experienced by individuals with CVD while viewing an image depends on their specific type of CVD, causing distinct details to vanish for individuals with protan or deutan deficiencies. Thus, calibration of the daltonization algorithm to the user’s CVD type, identified through the CAD method, is essential. For user convenience, implementing an autonomous CVD test to customize the algorithm according to the user’s condition would be advantageous.

Identifying CVD may be easier than specifying the precise disorder type (protan/deutan). Whereas Ishihara pseudoisochromatic plates rapidly detect color vision issues, they do not conclusively differentiate between protan and deutan deficiencies. Toufeeq addressed this concern in [59], but the suggested test is complex for home use. To our knowledge, there is no straightforward test in the English literature that accurately determines the type of CVD.

Thus, we implemented a test outlined by Maximov et al. [27]. It involves presenting three images: the original and simulations depicting how individuals with protanopia and deuteranopia perceive the image. These simulations are generated using Viénot’s algorithm [33]. Participants choose the image that varies the most in perceived color from the other two. Additionally, a “Not sure” option is available for ambiguous cases, accompanied by a text box to explain the decision.

Hypothetically, normal trichromats would choose the original image as significantly different, whereas dichromats would select a simulation representing the opposite type of CVD. For instance, protanopes would pick a simulation of deuteranopes’ perception, and vice versa. Subjective evaluation allowed this hypothesis to be tested, comparing the CVD express-test with the CAD test for dichromats, and revealing anomalous trichromat responses in the CVD express-test.

The CVD express-test used images randomly chosen from a set of 14 pre-selected images (Supplementary Figure S1).

To simplify pairwise comparisons, three images (the original and two simulations) were presented on the monitor screen at the corners of a triangle in a randomized sequence. Each participant made 14 selections, with a new triplet of images each time. The test involved 13 participants with varying degrees of CVD (refer to Supplementary Table S1), along with 3 normal trichromats.

### 6.5. CVD Express-Test Results

Supplementary Table S1 displays the outcomes of the CVD express-test and its agreement with the CAD test among CVD observers. Each participant’s red–green color discrimination thresholds from the CAD setup and their image selections in the CVD express-test are presented. The final column indicates the presumed diagnosis derived from the CVD express-test. The results for normal trichromats in the CVD express-test are excluded as they consistently and confidently distinguished the original full-color image from both simulations, as expected.

The table illustrates that individuals with CVD (both dichromats and anomalous trichromats) predominantly selected an opposing type of CVD simulation as the most distinct image, validating the diagnosis obtained via the CAD method. Protanopic dichromats exhibited a 100% match between the CVD express-test and the CAD test.

Unlike normal trichromats, anomalous trichromats rarely found the original (full-color) image to be the most distinct, often perceiving a simulation opposite to their CVD type as the most different. However, individuals with mild CVD had reduced accuracy in determining dichromacy type, as they often perceived the full-color image as distinct from both simulations.

Hence, the CVD express-test trials suggest its potential as an autonomous diagnostic tool for determining dichromacy type in individuals with CVD.

## 7. Results

The objective evaluation results for the proposed method, the anisotropic daltonization method [23], and the CVD-Swin method [28] are presented in Table 1 and Table 2. CVD-Swin is a recently introduced state-of-the-art neural network-based daltonization approach. Although pretrained models were not provided by the authors, the training framework is publicly available. Using this infrastructure, we trained two separate neural network models: one for protanopia and one for deuteranopia. A critical hyperparameter in the CVD-Swin framework is α, which controls the trade-off between naturalness preservation and contrast enhancement in the recolored images. While the original study explored values of α=0.25, 0.5, and 0.75, our experiments showed that at α=0.75, the model yields negligible contrast improvement. Therefore, we report and analyze results for models trained with α=0.25 and α=0.5.

**Figure 8:**
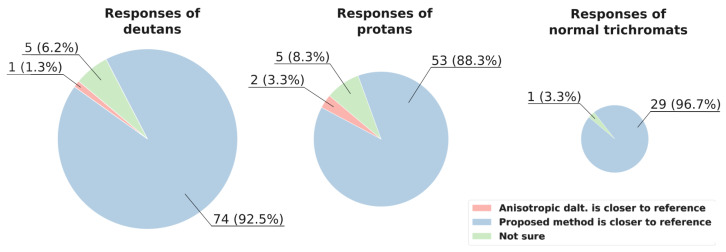
The naturalness assessment: human study results (selecting the image more similar to the reference). Each circle shows the total responses across all images for participants of the corresponding CVD type. The area of the circles is proportional to the number of participants of the corresponding CVD type.

**Figure 9:**
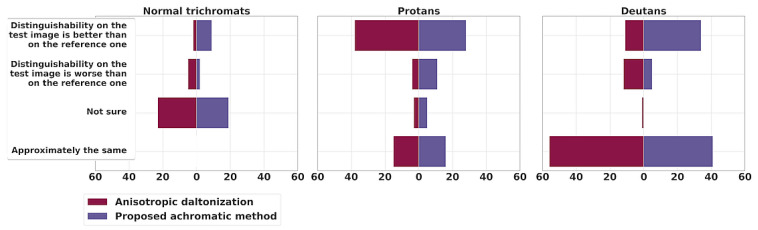
The contrast assessment: human study results (evaluating object distinguishability compared to the reference, without considering colors). Numbers on the diagrams indicate the sums of responses across all images for participants of the corresponding CVD type.

The subjective evaluation results of the proposed method and anisotropic daltonization method are presented in Figure 8 and Figure 9.

### 7.1. Qualitative and Objective Evaluation Results

**Figure 10:**
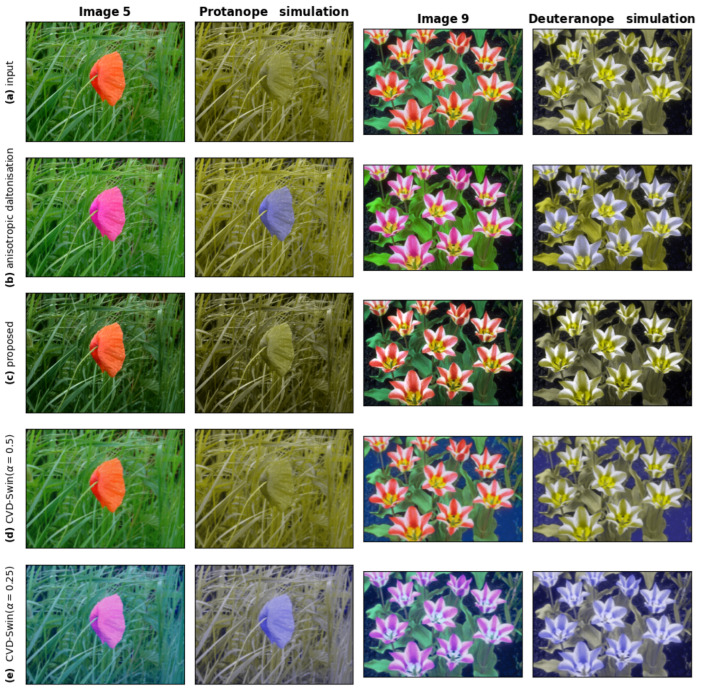
Images 5 and 9 processed for protanopes and deuteranopes, respectively. In rows: The original image, images derived using the anisotropic daltonization method, images obtained through the method proposed in our study, and images generated using CVD-Swin. Displayed in columns: The image and its corresponding simulation.

Figure 10 displays images processed for protanopia and deuteranopia. Additional figures are presented in Supplementary Figure S2. These figures showcase substantial improvements in differentiating flowers and fungi on their background, initially challenging for individuals with CVD, using both methods. However, in Supplementary Figure S2 (image 7), where the sun on the simulation image is nearly imperceptible without processing, only the proposed method fully restores its distinguishability for individuals with CVD. The anisotropic daltonization method, despite employing chromaticity distortion, fails to improve contrast in this specific area.

Table 1 displays the averaged naturalness preservation metrics ($CD_{Lab}$ (Formula (17)) and $CD_{proLab}$ (Formula (21)) calculated using the specified dataset (see Figure 7). The assessment included both cases for normal trichromats (shown in columns under “Mean original”) and for individuals with CVD (shown in columns under “Mean simulated”). The former utilizes the original image as the reference and the daltonized trichromatic image as the test image, whereas the latter employs simulations of both the original and daltonized images.

The proposed method consistently demonstrates lower CD values, highlighted in bold font, indicating closer chromaticity resemblance to the original image in all cases compared to the anisotropic method and CVD-Swin. Both metrics consistently favor our method across all instances, except for image 5 (Figure 10): the $CD_{Lab}$ metric implies that the anisotropic daltonization method produces less chromatically distinct results from the original compared to our algorithm (9.42 vs. 13.68 for deutans and 10.13 vs. 11.57 for protans). This contradicts visual perception, as shown in Figure 10. However, the $CD_{proLab}$ metric exhibits the opposite trend (0.0553 vs. 0.0159 for deutans and 0.0599 vs. 0.0125 for protans), aligning with the visual assessment.

Table 2 demonstrates contrast loss and restoration evaluations using the RMS metric. Unlike assessing naturalness, where the reference image changes depending on the observer’s CVD status, the original image consistently serves as the reference for contrast evaluation. The table includes a row labeled “No dalt.”, indicating contrast loss levels for protan and deutan simulations of the original image without any daltonization. On average, the anisotropic daltonization method surpasses the proposed method and CVD-Swin in restoring the original contrast for both types of CVD, according to the RMS metric. However, on average, the proposed achromatic method enhances contrast compared to the non-daltonized image.

To assess the influence of the selected optimization criterion and tone-mapping approach in the proposed method, an ablation study was conducted. The results indicate that the proposed configuration achieves the highest levels of both contrast and naturalness across most conditions. An exception is observed for the $CD_{proLab}$ metric in the case of deuteranopes, where the performance is comparable for both linear and non-linear optimization criteria (see Supplementary Table S2).

### 7.2. Subjective Evaluation Results

In Figure 8, the response statistics for naturalness are shown. Overall, the proposed achromatic method notably outperforms the anisotropic daltonization method in preserving naturalness.

Remarkably, in image 5 (Figure 10), where the $CD_{Lab}$ metric favored the anisotropic daltonization method, eight deutans and six protans unanimously selected our method’s output for higher naturalness, which corresponds to the results of $CD_{proLab}$.

When responding to the question about naturalness, participants experienced uncertainty, selecting “Not sure” for only two images: six participants (two deutans, three protans, and one trichromat) encountered difficulties with image 7 (represented in Figure 7), and six participants (three deutans, two protans, and one trichromat) had challenges with image 8 (represented in Figure 7). These two images, along with daltonizations for both CVD cases, are displayed in Supplementary Figure S3.

Let us move on to the question of the distinguishability of objects (Figure 9). The anisotropic daltonization method shows slightly more instances of distortion than improvement for deutans (11 cases of contrast improvement versus 12 cases of contrast loss). In 57 cases (“the same” in 56 cases and “not sure” in 1 case), the results were similar to the original contrast (“No dalt.”). Conversely, the achromatic algorithm performs better for deutans: enhancements were observed 34 times; in 5 instances, the images appeared worse after processing for deutans; and in 41 cases, participants noticed no change in comparison to the original contrast.

For protans, the anisotropic daltonization method performs notably better: in 38 instances, participants favored the processed images, whereas only in 4 cases did the contrast worsen compared to the original image. In 18 cases, no noticeable change in contrast was reported—15 responses indicated “the same”, and 3 were marked as “not sure”. In comparison, for the proposed method, 28 responses indicated enhanced contrast, 11 reported a reduction in contrast, 16 responses indicated “the same”, and 5 were “not sure”.

Thus, concerning contrast preservation in our dataset, the achromatic method yields better results than no processing, both for protans and deutans. In deutans’ responses the achromatic method outperforms the anisotropic daltonization method. Nonetheless, even for protans our method yields results not worse than no processing in 82% of responses, while surpassing the naturalness of the anisotropic daltonization method in 88% of responses.

Supplementary Figure S4 showcases the least successful outcomes of the proposed algorithm for protans, where some participants observed contrast loss for initially discernible objects.

It should be noted that the improvement in our method for normal trichromats is not unequivocal. Participants with normal color vision generally experienced difficulties in assessing contrast differences between processed and original images, regardless of the method used, and often perceived the processed image as barely distinguishable from the original.

To evaluate contrast improvement, we applied the sign test, grouping participants according to their type of CVD. Responses from normal trichromats were excluded from the analysis. Each participant evaluated 10 image pairs, resulting in a total of 80 responses from deutan participants (8 subjects × 10 images) and 60 from protan participants (6 subjects × 10 images). Responses were binarized: “Distinguishability on the test image is better than on the reference one” was coded as 1, and “Distinguishability on the test image is worse than on the reference one” as 0. Responses such as “Approximately the same” and “Not sure” were excluded from the analysis. The null hypothesis assumed a 50% chance of improved distinguishability (i.e., no effect). A two-tailed sign test was conducted at a significance level of α=0.05. The null hypothesis was rejected when the *p*-value fell outside the interval [0.025,0.975], indicating a statistically significant change in distinguishability. Additionally, we report the frequency of contrast improvement and deterioration—defined as the proportion of images for which participants indicated increased or decreased distinguishability, respectively, relative to the total number of images evaluated (including “Approximately the same” and “Not sure” responses). The results are summarized in Table 3. For the anisotropic daltonization method, deutan participants did not show a statistically significant improvement. Our method improved distinguishability in 49% of images and worsened it in only 6% for deutans. For protan participants, both methods showed similar rates of improvement.

## 8. Discussion

In this study, a novel daltonization method is introduced, preserving image naturalness for individuals with CVD and normal trichromats. The method achieves naturalness preservation by modifying solely the achromatic component of the image. Moreover, it enhances the distinguishability of image elements for dichromats by multiplying the input image by a coefficient map obtained through optimization aimed at preserving local contrast uniquely for each input image.

For the experimental investigation of the proposed method, a dataset of images was collected and published. Objective and subjective comparisons were conducted between our proposed method and the anisotropic daltonization method [23], since the latter also aims to preserve local contrast and is provided with a publicly available source code.

The subjective evaluation showed that the proposed method significantly outperforms the anisotropic daltonization in preserving naturalness for individuals with CVD and normal trichromats. The objective assessment employed both the established CD metric in the Lab coordinates and the proposed proLab modification of the CD metric. The modified metric better aligns with the participants’ responses. On average, both metrics indicated that the proposed method better preserves naturalness compared to the anisotropic daltonization.

Our findings corroborate the conclusions from the study in [7], indicating that traditional recoloring methods render colors unfamiliar and unattractive to individuals with CVD. Consequently, these tools are being utilized much more rarely than intended by their creators. As per the majority of participants in the survey [7], recoloring tools should minimize color alterations to align more closely with the colors that individuals with CVD are accustomed to perceiving.

Despite the fact that the proposed method can only modify the achromatic component, it turns out that in terms of contrast, it is superior to the anisotropic daltonization method for the surveyed deuteranopes. For protans, the proposed method is generally preferred over non-daltonized images and is planned to be further improved in future work.

The computational complexity of the proposed daltonization method, as well as that of the anisotropic daltonization method, is O(nM), where *n* denotes the number of pixels in the image and *M* represents the number of optimization iterations. In our experiments, the proposed method with *M* = 10,000 achieves a per-image processing time of 13.35 s. In comparison, the anisotropic daltonization method with M=500 requires 21.05 s for the same image. Both evaluations were conducted on a machine equipped with an Intel Core i7-11700F CPU at 2.50 GHz.

The main drawback of the proposed method is an insufficient level of contrast restoration in specific images: the human studies revealed instances where the achromatic daltonization did not sufficiently differentiate objects compared to the non-daltonized image. We attribute this to two factors. First, contrast assessment relies only on adjacent pixels, potentially causing ridge-like artifacts. However, as shown in Supplementary Figure S5, increasing the distance between points *p* and *q* in Equation (9) introduces visible artifacts into the output image. To mitigate this issue, future work will explore incorporating a broader pixel neighborhood in the contrast evaluation process. Second, some images show significant background darkening, which could be addressed using local tone mapping. We would like to emphasize that the aforementioned issues do not constitute fundamental limitations of the new approach, of which the method proposed in this paper is the first representative. The achromatic contrast-preserving approach, which maintains the image naturalness both for individuals with CVD and normal trichromats, warrants further development.

Additionally, we have validated a CVD express-test that allows for the determination of the type of dichromacy without the need for special equipment.

## Figures and Tables

**Table 1:** Averaged naturalness metrics (with standard deviations) for $CD_{Lab}$ and $CD_{proLab}$, with superior naturalness preservation indicated in bold font.

	${\mathrm{CD}}_{\mathrm{Lab}}$		${\mathrm{CD}}_{\mathrm{proLab}}$	
	Mean Original	Mean Simulated	Mean Original	Mean Simulated
	**Deutans**			
Anisotropic	14.33 ± 5.88	13.98 ± 6.17	0.1234 ± 0.0540	0.1164 ± 0.0514
Proposed	**6.36 ± 4.74**	**4.31 ± 3.0**	**0.0138 ± 0.0091**	**0.0090 ± 0.0057**
CVD-Swin (α = 0.5)	12.22 ± 6.94	11.07 ± 7.86	0.1456 ± 0.1005	0.1216 ± 0.0982
CVD-Swin (α = 0.25)	32.58 ± 6.82	33.15 ± 7.62	0.3197 ± 0.1007	0.2994 ± 0.0943
	**Protans**			
Anisotropic	15.72 ± 6.61	15.54 ± 6.57	0.1346 ± 0.0584	0.1352 ± 0.0562
Proposed	**5.86 ± 3.0**	**4.40 ± 2.45**	**0.0118 ± 0.0047**	**0.0074 ± 0.0035**
CVD-Swin (α = 0.5)	10.1 ± 5.35	9.22 ± 5.61	0.1192 ± 0.0842	0.1005 ± 0.0753
CVD-Swin (α = 0.25)	33.18 ± 12.18	32.78 ± 12.11	0.3279 ± 0.1254	0.2978 ± 0.1137

**Table 2:** The RMS calculations on simulated images using the anisotropic daltonization and our achromatic method. Variants with superior original contrast preservation, as indicated by RMS, are highlighted in bold font.

RMS											
**Deutan**	**1**	**2**	**3**	**4**	**5**	**6**	**7**	**8**	**9**	**10**	**Mean**
No dalt.	0.1182	0.0742	0.1218	0.1361	0.1108	0.0321	**0.0179**	0.0929	0.1169	0.0951	0.0916
Anisotropic	**0.0748**	**0.0695**	0.0955	0.1291	0.1068	0.0446	0.0545	0.1123	**0.0989**	**0.0823**	**0.0868**
Proposed	0.1166	0.0841	0.1140	0.1396	0.0914	**0.0311**	0.0435	**0.0633**	0.1199	0.0998	0.0903
CVD-Swin (α = 0.5)	0.1379	0.0808	**0.0588**	0.1294	0.1115	0.0703	0.0287	0.0899	0.1310	0.1423	0.0981
CVD-Swin (α = 0.25)	0.0958	0.0748	0.1146	**0.1218**	**0.0777**	0.1122	0.0591	0.0722	0.1354	0.1101	0.0973
**Protan**	**1**	**2**	**3**	**4**	**5**	**6**	**7**	**8**	**9**	**10**	**Mean**
No dalt.	0.1243	0.0956	0.1922	0.1491	0.1443	**0.0272**	**0.0388**	0.1390	0.1415	0.1034	0.1155
Anisotropic	**0.0347**	**0.0478**	**0.0438**	**0.0971**	**0.0514**	0.0385	0.0409	**0.0846**	**0.0886**	**0.0451**	**0.0573**
Proposed	0.1447	0.0969	0.1818	0.1616	0.1358	0.0289	0.0404	0.0960	0.1456	0.1119	0.1144
CVD-Swin (α = 0.5)	0.0833	0.0946	0.1510	0.1439	0.1533	0.0671	0.0416	0.1407	0.1446	0.1358	0.1156
CVD-Swin (α = 0.25)	0.0903	0.0683	0.0888	0.1482	0.0915	0.1007	0.0518	0.1028	0.1279	0.1143	0.0985

**Table 3:** Results of the sign test assessing object distinguishability for participants with CVD. N indicates the number of binary responses comparing object distinguishability between the test and reference images (i.e., whether it was “better” (Increased) or “worse” (Decreased) on the test image). *p*-values corresponding to statistically significant results are highlighted in bold.

CVD	Anisotropic Method				Proposed Method			
	N	*p*-Value	Increased	Decreased	N	*p*-Value	Increased	Decreased
Deutans	23	0.500	0.29	0.15	39	**1.000**	0.49	0.063
Protans	42	**1.000**	0.70	0.067	39	**0.998**	0.65	0.18

## Data Availability

The collection of images used to evaluate the daltonization methods, along with the subjective evaluation results presented in this study, is openly available at https://zenodo.org/records/14170170 (accessed on 24 June 2025).

## Supplementary Materials

The following supporting information can be downloaded at: https://www.mdpi.com/article/10.3390/jimaging11070225/s1, Figure S1: Image set used in the color vision deficiency (CVD) express-test; Figure S2: Images 2 and 7 processed for protanopia (columns show: the original image, images processed using the anisotropic daltonization method, and images processed using the method proposed in this study; rows show: the image and its corresponding simulation); Figure S3: Examples of two images (8 and 7) where participants had difficulty selecting the image most similar to the reference («No dalt.»); Figure S4: Examples of processing for individuals with protanopia, where, according to some participants, the contrast in the achromatic method appears degraded compared to the original (columns show: the original image, a simulation of the original, and the image processed using the proposed method); Table S1: Results of the express-test conducted for participants with color vision deficiency (CVD); Figure S5: Example images (5 and 6) illustrating how increasing the distance between points *p* and *q* in Equation (15) results in the appearance of grid-like artifacts. Table S2: Ablation study results showing the impact of different optimization criteria and tone-mapping strategies on objective evaluation metrics.

## Abbreviations

The following abbreviations are used in this manuscript:

CVD	Color vision deficiency
L	Long
M	Medium
S	Short
CIE	International Commission on Illumination
NCV	Normal color vision
CAD	Color assessment and diagnosis
RMS	Root mean square
CD	Chromatic difference
