# Supplementary material for: Leveraging Achromatic Component for Trichromat-Friendly Daltonization
Source: J Imaging. 2025 Jul 7;11(7):225. doi: 10.3390/jimaging11070225 (PMC12295553; doi:10.3390/jimaging11070225)
Supplement: Supplementary file 1 [file jimaging-11-00225-s001.zip › jimaging-3698662-supplementary.pdf]

Leveraging Achromatic Component for Trichromat-Friendly Daltonization: supplemental document

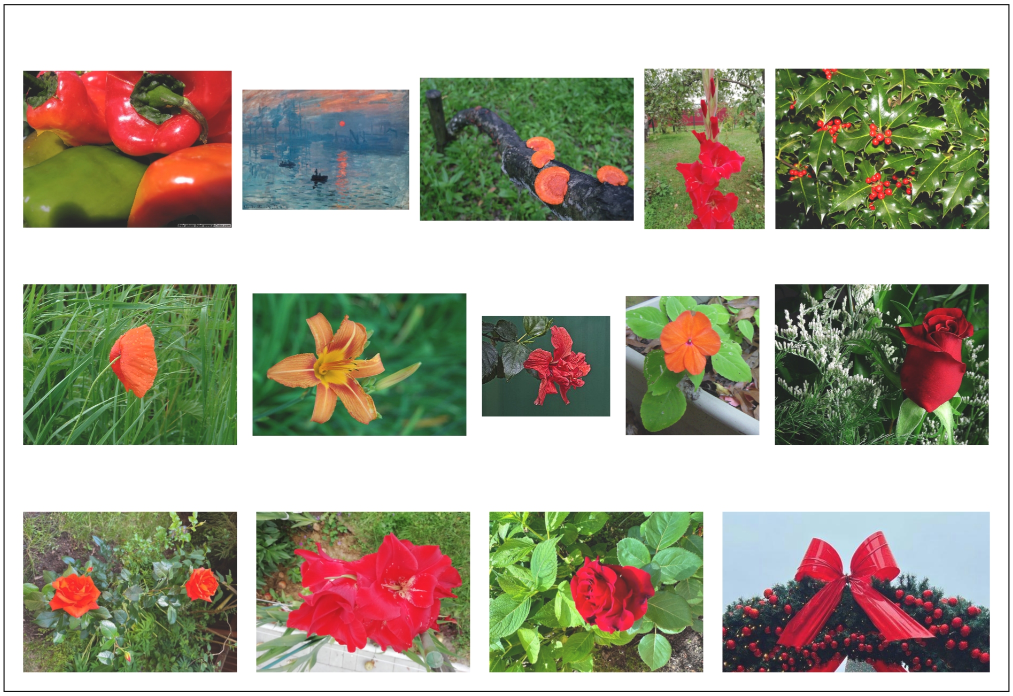

Figure S1. Image set used in the Color Vision Deficiency (CVD) express test

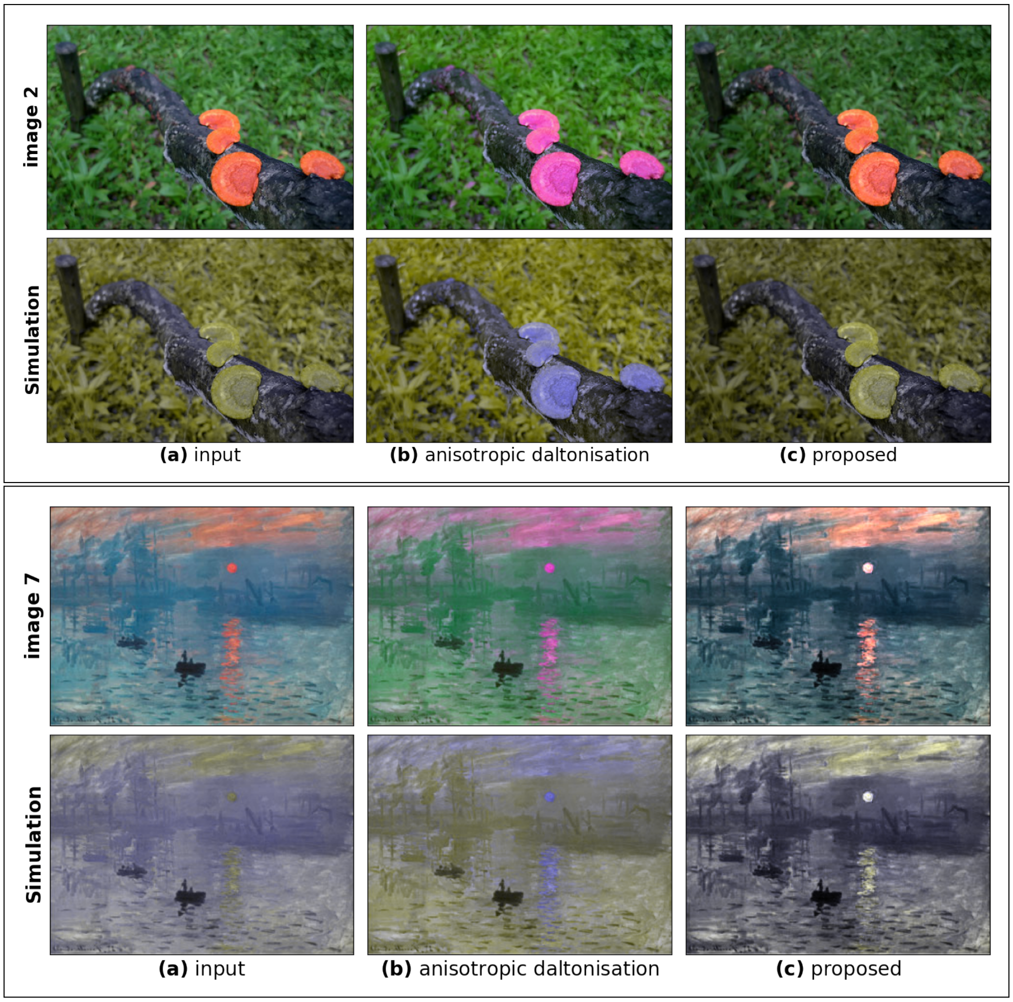

Figure S2. Images 2 and 7 processed for protanopia (columns show: the original image, images processed using the anisotropic daltonization method, and images processed using the method proposed in this study; rows show: the image and its corresponding simulation).

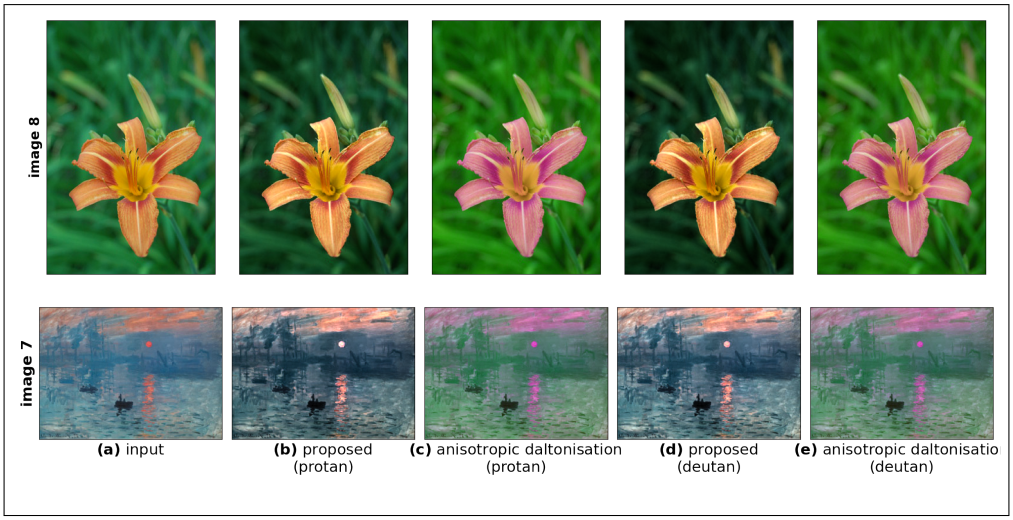

Figure S3. Examples of two images (8 and 7) where participants had difficulty selecting the image most similar to the reference («No dalt.»).

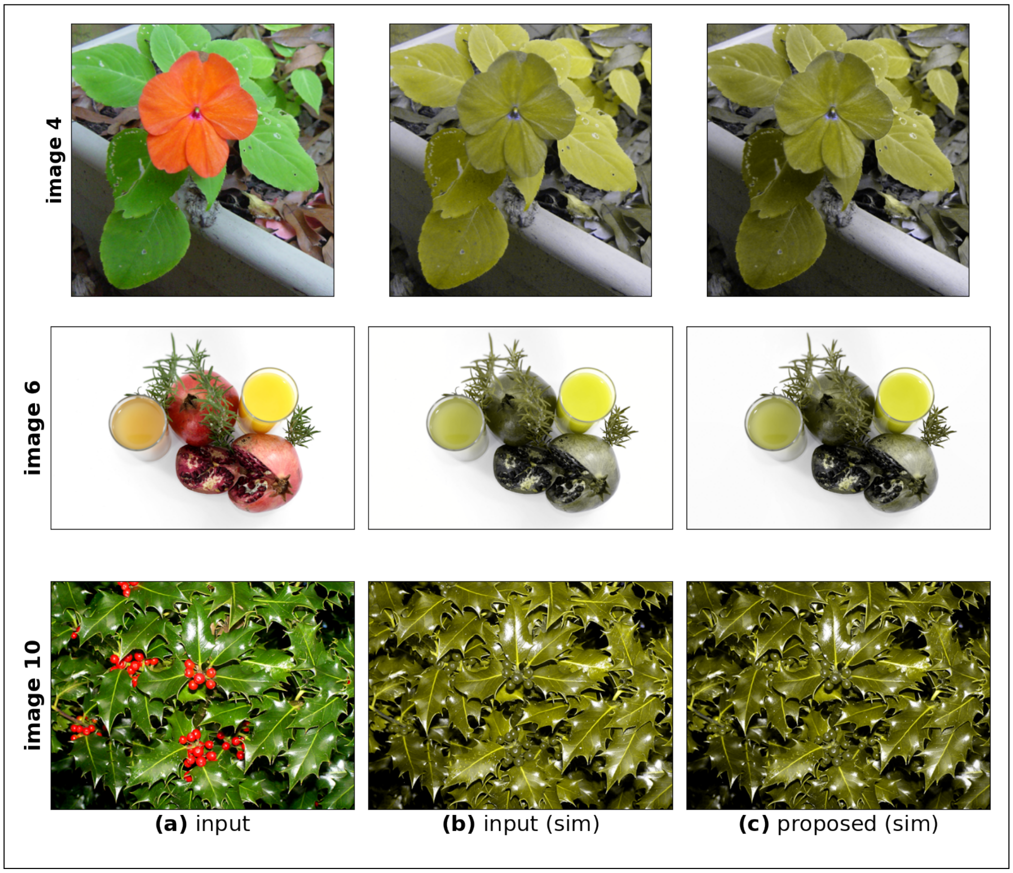

Figure S4. Examples of processing for individuals with protanopia, where, according to some participants, the contrast in the achromatic method appears degraded compared to the original (columns show: the original image, a simulation of the original, and the image processed using the proposed method).

Table S1. Results of the express test conducted for participants with Color Vision Deficiency (CVD).

ID	RG threshold (CAD units)	Number of protanopia simulations selected	Number of deuteranopia simulations selected	Number of original pictures selected	Test result
Protanopes					
BA	30.7	0	14	0	Protan
UG	29.5	0	14	0	Protan
NA	26.5	0	14	0	Protan
Protanomalous trichromat					
BD	25.5	0	14	0	Protan
CU	24.0	0	11	0	Protan
RD	22.7	1	11	2	Protan
Deuteranopes					
MS	30.0	13	0	0	Deutan
Deuteranomalous trichromat					
IG	21.3	11	0	3	Deutan
HD	21.3	12	1	1	Deutan
PD	19.7	13	1	0	Deutan
LI	18.5	14	0	0	Deutan
KN	16.7	11	3	0	Deutan
AI	13.5	4	1	9	Normal/ Deutan

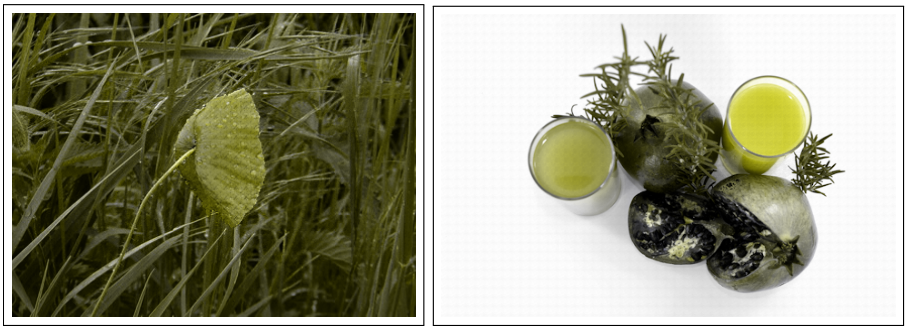

Figure S5. Example images (5 and 6) illustrating how increasing the distance between points p and q in equation (15) results in the appearance of grid-like artifacts.

Table S2. Ablation study results showing the impact of different optimization criteria and tone mapping strategies on objective evaluation metrics.

	RMS	CD_Lab		CD_proLab	
		Mean original	Mean simulated (protan)	Mean original	Mean simulated (protan)
Proposed method	0.1144	5.86	4.40	0.0118	0.0074
Proposed method with linear criterion	0.1218	6.2	4.74	0.0131	0.0074
Proposed method with normalization to maximum as tone mapping	0.1307	9.87	7.35	0.0195	0.0122

	RMS	CD_Lab		CD_proLab	
		Mean original	Mean simulated (deutan)	Mean original	Mean simulated (deutan)
Proposed method	0.0928	5.21	3.64	0.0117	0.0074
Proposed method with linear criterion	0.1001	5.3	3.8	0.0115	0.0071
Proposed method with normalization to maximum as tone mapping	0.1079	9.27	6.68	0.0181	0.0117
